# Neurofibromas on the Hands

**Published:** 2018-03-19

**Authors:** Joseph Behrens, Dave S. K. Ho, Gianfranco Frojo, Kashyap K. Tadisina, Bruce A. Kraemer

**Affiliations:** Division of Plastic Surgery, Saint Louis University School of Medicine, St Louis, Mo

**Keywords:** hand mass, neurofibroma, neurofibromatosis, schwannoma, second palmar web space

**Figure:**
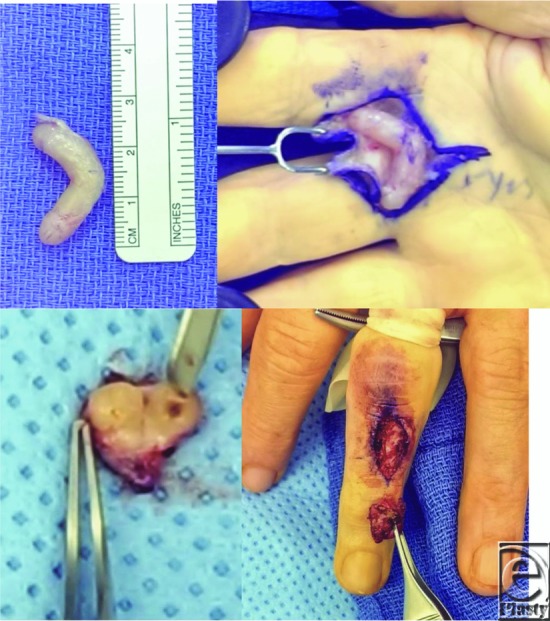


## DESCRIPTION

A 54-year-old right-hand-dominant man presented with a 2-year history of growing, painful masses that when excised measured 1×1 cm on the dorsal aspect of the left middle phalanx and 3×0.7 cm on the second palmar web space of the right hand. Pathology was consistent with neurofibromas.

## QUESTIONS

1. How will a patient with multiple neurofibromas in the hands first present in the clinical setting?
2. What modalities facilitate the evaluation of a suspected neurofibroma?
3. How are neurofibromas distinguished from schwannomas in practice, as both are neural sheath tumors?
4. What are indications and possible complications considered for surgical excision of a neurofibroma?

## DISCUSSION

Neurofibromas are diffuse proliferations of cells derived from neural and mesenchymal origins that may occur sporadically or in the setting of neurofibromatosis.^1^ Most cases of multiple neurofibromas are associated with neurofibromatosis type 1, an autosomal dominant multisystem disease due to a mutation of the homonymous tumor-suppressor gene located on chromosome 17.^2^ Patients with this condition can present with cutaneous, spinal, or plexiform neurofibromas.^3^

In the hand, neurofibromas tend to arise near flexion creases and cutaneous nerves.^1^ Cutaneous neurofibromas are most commonly identified after excision of a solitary lesion in adults with no family history of neurofibromatosis. These hand masses may present in the clinic as flesh-colored or potentially hyperpigmented papular nodules that cause varying degrees of localized pain or peripheral nerve dysfunction.^1^ Although Boyd et al^3^ describe a technique where pressure is applied to invaginate the lesion and reappearance is expected upon release, this maneuver is less likely to be positive in the hand due to the unyielding fascia of the glabrous palm and extensor tendons on the dorsal surface.

Magnetic resonance imaging (MRI) is the first-line imaging modality for distinguishing neurofibromas from other common hand masses. “Target sign” findings describe the peripherally enhancing rim and hypointense central regions on T2-weighted magnetic resonance image (T2w).^4^ Clinicians must also take into consideration ganglion cysts, as they are the most common hand masses that appear smooth, well-circumscribed, and noninvasive on T1-weighted magnetic resonance image (T1w) and solid hyperintense masses on T2w. Epidermal cysts also appear as a well-circumscribed lesion but can be distinguished by a fluid sign in the dermis or subdermis on T1w. On T2w, epidermal cysts will vary depending on internal keratin content. Fibromas of the tendon sheath are well differentiated with a low signal on T1w and a varied heterogeneous enhancement on T2w, indicating the varying proportions of cellular and fibrous tissue. Finally, lipomas are easily identifiable on magnetic resonance image as hyperintense well-circumscribed lesions.^5^

While MRI is a powerful imaging modality used to distinguish neurofibromas from an assortment of hand masses, it cannot differentiate between other nerve sheath tumors such as schwannomas. Other imaging modalities such as ultrasonography and computed tomography (CT) also fail to further narrow the diagnosis. Both neurofibromas and schwannomas appear as well-defined, encapsulated masses with hypoechoic features on ultrasound scan and are hypodense on CT scan.^6^ Histopathology is the only definitive way to identify a neurofibroma from a schwannoma and nerve sheath tumors stain for S-100. Neurofibromas are consistent with embryological origins and have a loose, myxomatous background with low cellularity. Schwannomas grow in Antoni type A and B patterns. Type A is a cellular growth pattern in stromal matrix with whorled proliferation of the nerve sheath, whereas type B is a myxoid matrix with low cellularity and vacuolar degeneration.^7^

Surgery is indicated for lesions greater than 4 cm or when the suspected neurofibromas cause pain, numbness, or limited range of motion. When located near major peripheral nerves, surgical intervention is offered earlier both to relieve the discomfort and to accurately differentiate it from a schwannoma involving the nerve.^1^^,^^8^ In this operative case, one of the lesions located within the right first volar web space was identified on intraoperative dissection. The lesion was located adjacent to the flexor digitorum superficialis tendons of the index and the long finger. The left hand mass was located on the dorsal aspect of the long finger middle phalanx. During dissection, both masses were found to be of similar size and in close proximity to respective digital arteries but did not involve the digital nerves.
